# Development of a Wearable-Sensor-Based Fall Detection System

**DOI:** 10.1155/2015/576364

**Published:** 2015-02-16

**Authors:** Falin Wu, Hengyang Zhao, Yan Zhao, Haibo Zhong

**Affiliations:** ^1^School of Instrumentation Science and Optoelectronics Engineering, Beihang University, Beijing 100191, China; ^2^Space Star Technology Co., Ltd., Beijing 100086, China

## Abstract

Fall detection is a major challenge in the public healthcare domain, especially for the elderly as the decline of their physical fitness, and timely and reliable surveillance is necessary to mitigate the negative effects of falls. This paper develops a novel fall detection system based on a wearable device. The system monitors the movements of human body, recognizes a fall from normal daily activities by an effective quaternion algorithm, and automatically sends request for help to the caregivers with the patient's location.

## 1. Introduction

Falls of the elderly always lead to serious health issues as the decline of their physical fitness [1]. Fracture is the most common injury in fall of an elderly and there is also a certain possibility to get coma, brain trauma, and paralysis. At most fall situations, the fall process is the main source of injury because of the high impact. But sometimes the late medical salvage may worsen the situation [2]. That means the faster the salvage comes, the less risk the elderly will face.

Progress of technology brings more possibilities to help us protect the elderly. Low power consumption components make it possible to realize wearable monitoring device. MEMS (microelectro mechanical systems) sensors have simplified the design and implementation of sensor system. Location based service (LBS) makes it more convenient to locate the elderly in health monitoring. Beside these, mobile computing makes remote health monitoring easier to realize.

Several kinds of fall detection methods have been developed or applied in our life [3]. One of them is computer vision based method. Cameras are distributed at limited space to offer pictures or videos of human activities to implement fall detection algorithm. External supports such as motion sensors could be used to enhance computer vision based fall detection method [4], and a data fusion algorithm can operate the validation and correlation among the two subsystems to raise robust performance of fall detection. These computer based methods work effectively in indoor environment, but they are hard to realize in outdoor environment as the deployment of cameras is always limited.

Motion sensor-based method is also commonly used. Accelerometer and gyroscope could provide linear and angular motion information directly. Sensor measurements or their proper fusion could be used to distinguish a real fall. There are several kinds of detection methods which differ in constitution of motion sensors and detection algorithms. The first kind of detection method is using an accelerometer. A single triaxial accelerometer can provide object's accelerations in three directions which include the influence of gravity. A coordinate will be built when the accelerometer is fixed on human's body. The influence of gravity or dynamic acceleration is available by using a low pass filter or a high pass filter [5]. Some kinds of angular movement information can also be calculated based on the relationship between acceleration components and their vector sum [6]. The second kind of detection method is based on both accelerometer and gyroscope [7]. Gyroscope can offer angular velocity and the accelerometer could offer linear motion information. The third kind of detection method also uses a magnetometer. A triaxial magnetometer can detect magnetic strength in three directions, and it can also provide angular motion information in the horizontal plane. But the environment magnetic field may disturb the geomagnetic field which reduces the reliability of the magnetometer outputs, for instance, in some steel structure architecture or near some objects with strong electromagnetism. As angular information can also be extracted from accelerometer measurements, a state space filter such as the Kalman filter is a commonly used technique to combine angular motion information [8]. Beside these, sensors such as barometer can also assist pure motion sensors at human gait recognition [9].

But, in fact, using more sensors means more power consumption, and it is a challenge to design a proper algorithm to fuse different kinds of sensors. A single triaxial accelerometer is quite enough for human fall detection as sufficient information could be extracted from its measurements. Besides this, the accelerometer coordinate does not have to be fixed if only the magnitude of sum vector is needed [10], and that is quite convenient for wearable application. In this paper, a fall detection system based on a wearable device is developed. The hardware and software realization of the device is mainly based on a single triaxial accelerometer and GPS/GSM module. The device uses an efficient fall detection algorithm with less resource and power consumption, which means that it is a proper design for outdoor application.

## 2. System Design

**Figure 1:**
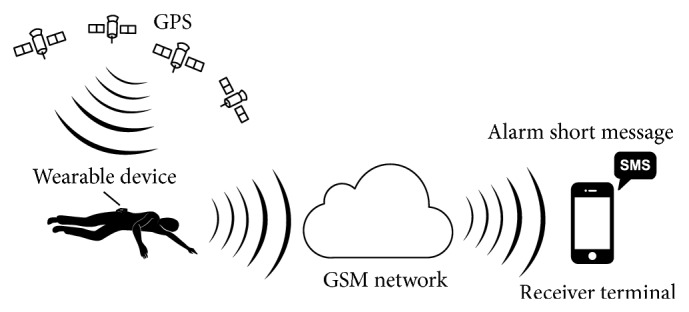
System architecture.

The architecture of the developed system is described in Figure 1. A wearable device is placed on human's waist. The system can detect the elderly's falling by acceleration analysis. Then it will get the elderly's geographic position and send fall alarm short message to caregivers. So the elderly who has fallen can get timely help to minimize the negative influence.

## 3. Fall Detection Algorithm

### 3.1. Overview

Choice of recognition feature has decisive significance to successful fall detection. Information like linear movements (e.g., displacement, velocity, and acceleration) and angular movements (e.g., angle, angular velocity, and angular acceleration) could be obtained directly or indirectly. Beside these, frequency domain parameters could be extracted from basic sensor measurements by techniques such as FFT and wavelet [11, 12]. For single triaxial accelerometer application, accelerations and derived angular parameters could be used as recognition features.

Fall detection algorithm design is based on the choice of recognition features. According to the recognition feature, fall detection algorithms are classified as threshold based and machine learning based. For threshold based method, threshold of recognition feature is set by the designer before application which makes the algorithm have rapid response and less resource consumption [13]. But the choice of threshold needs both rigorous schemes and adequate experiments. For machine learning based design, the classification of fall and normal activities is available with the assistance of technologies such as support vector machine (SVM) and neural network [14, 15]. Machine learning assistance may enhance system robustness to some extent, but its algorithm design is always high computing resource consumed which limits its application in wearable device. As the compact wearable device requires low power consumption and a single triaxial accelerometer could provide effective information, threshold based fall detection algorithm will be used in this system.

### 3.2. Algorithm Design

Algorithm used in this fall alarm system is based on thresholds of sum acceleration and rotation angle information. When a real fall happens, collision between human's body and ground will produce obvious peak value at the sum acceleration **a** which has magnitude as

(1) $$\begin{array}{c} \left|a\right|=\sqrt{{a_{x}}^{2}+{a_{y}}^{2}+{a_{z}}^{2}}, \end{array}$$

where *a*_*x*_, *a*_*y*_, and *a*_*z*_ present accelerometer measurements of three axes. The system uses the sum acceleration as the first step to distinguish high intensity movements from others. But normal motions such as jumping or sitting also produce peak values, which mean that additional detection features are required.

**Figure 2:**
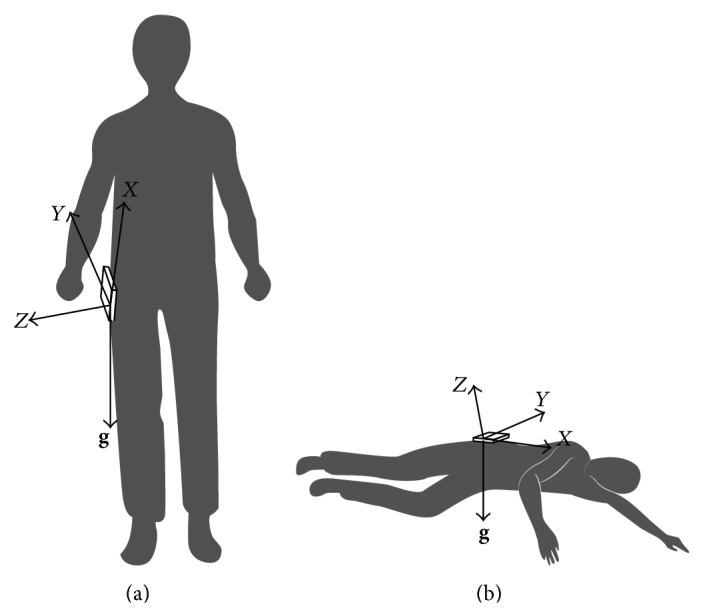
Coordinate and gravity before and after falling. (a) Before falling. (b) After falling.

**Figure 3:**
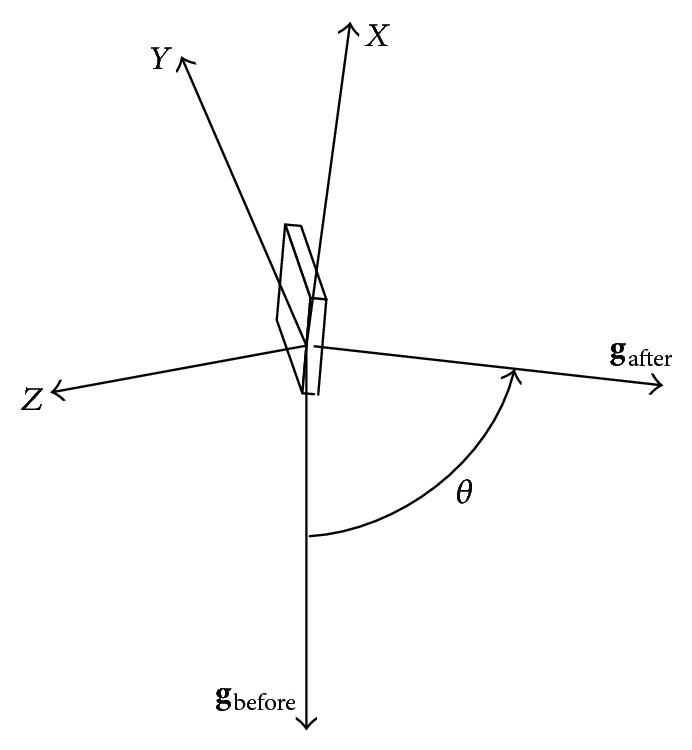
Rotation of gravity relative to fixed coordinate.

The second feature used here is an angle calculated based on acceleration measurements. As human's motion has low acceleration, it is feasible to get gravity component in each axis by using a low pass filter. If gravity components could be separated before and after human's fall, then it is possible to calculate the rotation angle of accelerometer coordinate in 3D space, which is also equivalent to the rotation angle of gravity vector relative to fixed coordinate. Coordinate constructed by the accelerometer and the gravity vector is shown in Figure 2. The rotation of gravity vector in fixed coordinate is shown in Figure 3.

Quaternion is an effective tool to describe rotation movement in human's gait change which also includes falling [16]. A quaternion could be described as

(2) $$\begin{array}{c} Q=q_{0}+q_{1}\cdot \overset{-}{i}+q_{2}\cdot \overset{-}{j}+q_{3}\cdot \overset{-}{k} \end{array}$$

which has magnitude as

(3) $$\begin{array}{c} \left\|Q\right\|=\sqrt{{q_{0}}^{2}+{q_{1}}^{2}+{q_{2}}^{2}+{q_{3}}^{2}}. \end{array}$$

Unit quaternion which has magnitude ‖**Q**‖ = 1 can be described as

(4) $$\begin{array}{c} Q=\cos\frac{\theta }{2}+\sin\frac{\theta }{2}\cdot q_{x}\cdot \overset{-}{i}+\sin\frac{\theta }{2}\cdot q_{y}\cdot \overset{-}{j}+\sin\frac{\theta }{2}\cdot q_{z}\cdot \overset{-}{k}. \end{array}$$

As shown in Figure 3, rotation angle of **Q** equals *θ*. The rotation axis is orthogonal to the rotation plane and its direction is in accordance with right hand screw rule. *q*_*x*_, *q*_*y*_, and *q*_*z*_ are three components of the unit vector which describes the orientation of the quaternion at the fixed coordinate.

Besides rotation movement, quaternion can also describe a vector in 3D space, such as the gravity vector **g** which could be described as a quaternion

(5) $$\begin{array}{c} g=0+g_{x}\cdot \overset{-}{i}+g_{y}\cdot \overset{-}{j}+g_{z}\cdot \overset{-}{k}. \end{array}$$

*g*_*x*_, *g*_*y*_, and *g*_*z*_ are three components of **g** which has quaternion magnitude as

(6) $$\begin{array}{c} \left\|g\right\|=\sqrt{g_{x}^{2}+g_{y}^{2}+g_{z}^{2}}=g. \end{array}$$

**Figure 4:**
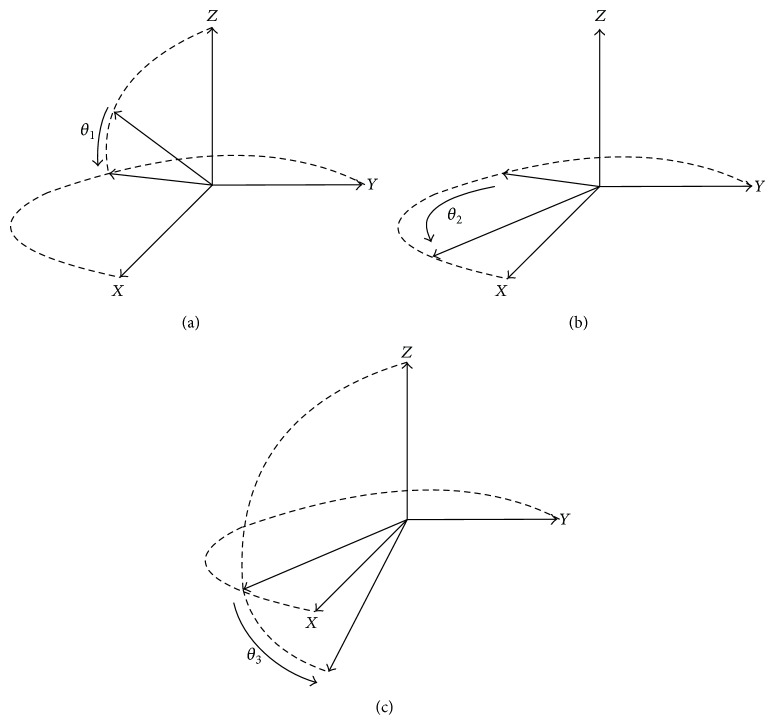
Decomposition of fall rotation quaternion **Q**. (a) Quaternion **Q**_1_. (b) Quaternion **Q**_2_. (c) Quaternion **Q**_3_.

A unit quaternion **Q** is used to describe human's falling movement, which can also be divided into three rotation quaternions **Q**_1_, **Q**_2_, and **Q**_3_ to simplify the calculation as shown in Figure 4.

With the help of gravity vector information before and after human's falling movement as shown in Figure 3, these three separated quaternions are all available.

**Q**_1_ could be expressed as

(7) $$\begin{array}{c} Q_{1}=\cos\frac{\theta_{1}}{2}+\sin\frac{\theta_{1}}{2}\sin\alpha \cdot \overset{-}{i}+\sin\frac{\theta_{1}}{2}\cos\alpha \cdot \overset{-}{j} \end{array}$$

which has rotation angle and rotation axis information as

(8) $$\begin{array}{c} \theta_{1}=\arctan\frac{g_{\text{before},z}}{\sqrt{g_{\text{before},x}^{2}+g_{\text{before},y}^{2}}},\\ \sin\alpha =\frac{-g_{\text{before},y}}{\sqrt{g_{\text{before},x}^{2}+g_{\text{before},y}^{2}}},\\ \cos\alpha =\frac{g_{\text{before},x}}{\sqrt{g_{\text{before},x}^{2}+g_{\text{before},y}^{2}}}. \end{array}$$

Quaternion **Q**_2_ can be calculated as

(9) $$\begin{array}{c} Q_{2}=\cos\frac{\theta_{2}}{2}+\sin\frac{\theta_{2}}{2}\cdot \overset{-}{k} \end{array}$$

which has rotation angle as

(10) $$\begin{array}{c} \theta_{2}=\arctan2\frac{g_{\text{after},y}}{g_{\text{after},x}}-\arctan2\frac{g_{\text{before},y}}{g_{\text{before},x}}. \end{array}$$

Quaternion **Q**_3_ is

(11) $$\begin{array}{c} Q_{3}=\cos\frac{\theta_{3}}{2}+\sin\frac{\theta_{3}}{2}\sin\beta \cdot \overset{-}{i}+\sin\frac{\theta_{3}}{2}\cos\beta \cdot \overset{-}{j} \end{array}$$

with rotation angle and rotation axis information as

(12) $$\begin{array}{c} \theta_{3}=-\arctan\frac{g_{\text{after},z}}{\sqrt{g_{\text{after},x}^{2}+g_{\text{after},y}^{2}}},\\ \sin\beta =\frac{-g_{\text{after},y}}{\sqrt{g_{\text{after},x}^{2}+g_{\text{after},y}^{2}}},\\ \cos\beta =\frac{g_{\text{after},x}}{\sqrt{g_{\text{after},x}^{2}+g_{\text{after},y}^{2}}}. \end{array}$$

Then the rotation of the fall movement can be expressed by quaternion multiplication as

(13) gafter=Q⊗gbefore⊗Q∗=Q3⊗Q2⊗Q1⊗gbefore⊗Q1∗⊗Q2∗⊗Q3∗,

where $Q^{*}=q_{0}-q_{1}\cdot \overset{-}{i}-q_{2}\cdot \overset{-}{j}-q_{3}\cdot \overset{-}{k}$ is the conjugate quaternion of **Q**. Quaternion algebra is normally implemented based on basic matrix algebra [17]. Quaternion multiplication used above could be realized by matrix multiplication as

(14) Q⊗gbefore=MQ0gbefore,xgbefore,ygbefore,z=q0−q1−q2−q3q1q0−q3q2q2q3q0−q1q3−q2q1q00gbefore,xgbefore,ygbefore,z.

The whole rotation quaternion can also be decomposed as

(15) Q=Q3⊗Q2⊗Q1=MQ3MQ2Q1.

**Q**_1_, **Q**_2_, and **Q**_3_ are all available based on gravity information before and after the falling movement. At last, it is possible to get four elements of quaternion **Q** by the equation above and the rotation angle *θ* could be calculated as

(16) $$\begin{array}{c} \theta =2\cdot \arctan\left(\frac{\sqrt{{q_{1}}^{2}+{q_{2}}^{2}+{q_{3}}^{2}}}{q_{0}}\right). \end{array}$$

When an object is falling, magnitude of *θ* will approach 90° which is also a character different from most normal activities. So it could be used as the second detection feature beside sum acceleration.

**Figure 5:**
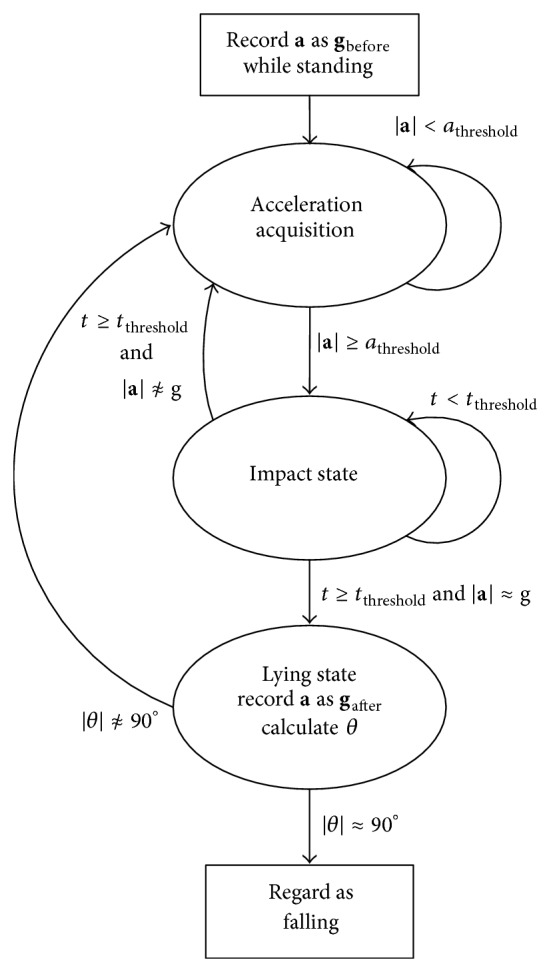
Fall detection algorithm state machine.

The flow chart of the proposed algorithm is depicted in Figure 5.

The wearable device will be mounted on human's waist at first to reflect the motion of human body closely [18], and the device will record **g**_before_ while the elderly is standing still. There is no special requirement of the device orientation but only keep stationary during the wear. *a*_threshold_ means the threshold of sum acceleration magnitude and *t*_threshold_ means the threshold of oscillation time duration after the break of *a*_threshold_.

When measurements in three different axes have been acquired, the sum acceleration |**a**| will be calculated. When a real fall happens, sum acceleration will reach peak value of |**a**| ≥ *a*_threshold_. In a real falling, the fluctuation of acceleration will stop in time duration *t*_threshold_ and then the sum acceleration is |**a**| ≈ *g* as the elderly will lie on the ground. Then the acceleration **a** is recorded as **g**_after_. Quaternion rotation *θ* will be calculated based on information **g**_before_ and **g**_after_. At last, the system will consider it as a fall if the rotation angle |*θ*| ≈ 90°.

## 4. Implementation

### 4.1. Hardware

ADI's digital triaxial accelerometer ADXL345 is the motion sensor used in this system. The GPS service and GSM communication function are integrated in SIMCom's SIM908 module. TI's 16 bits MCU MSP430F1611 is used to control the whole system and imply the detection algorithm [19].

The measurement range of accelerometer could be set at ±2 g, ±4 g, ±8 g, or ±16 g, and the maximal sampling rate is 3.2 kHz. As human's activities are normally at low frequency bands [20], 100 Hz is a proper sampling rate for human fall detection. There is an inner digital filter in ADXL345 which could weaken noise and reduce the burden of digital signal processing in MCU to some extent. The measurements will be sent through IIC (interintegrated circuit) bus communication between the sensor and the MCU.

SIM908 can offer GPS and GSM service on serial port communication with MCU, and it can also work in low power mode.

Each hardware component of the wearable device is working under low voltage and the detection algorithm does not need complex calculation resource, so the power consumption of the whole device is quite low. A 1200 mAh, 3.7 V polymer lithium battery is quite enough to provide the need of the wearable device for a couple of days.

**Figure 6:**
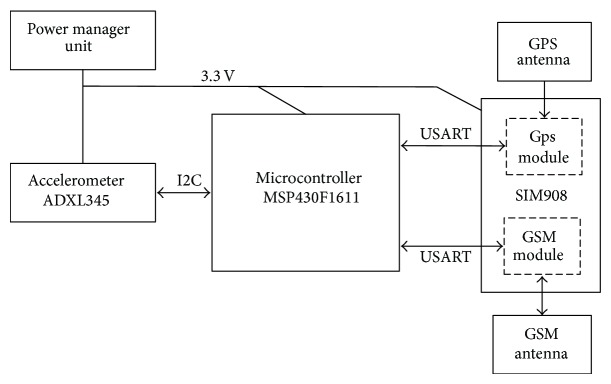
Basic hardware structure.

**Figure 7:**
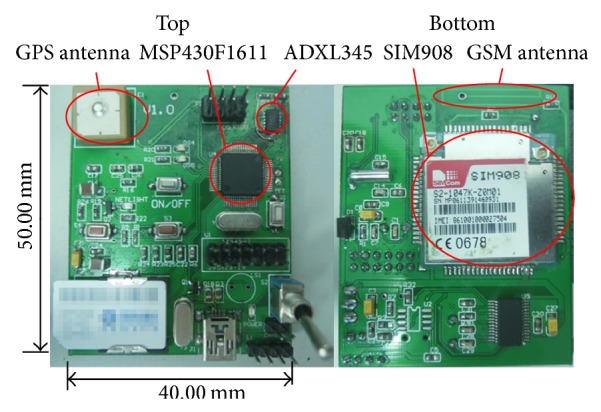
PCB board prototype.

Hardware structure of the detection device is shown in Figure 6, and the PCB board prototype is shown in Figure 7.

### 4.2. Software

The software design of the system can be divided into two parts. One of them is the software design in wearable device, and the other is in the caregiver's handset.

#### 4.2.1. Wearable Device

**Figure 8:**
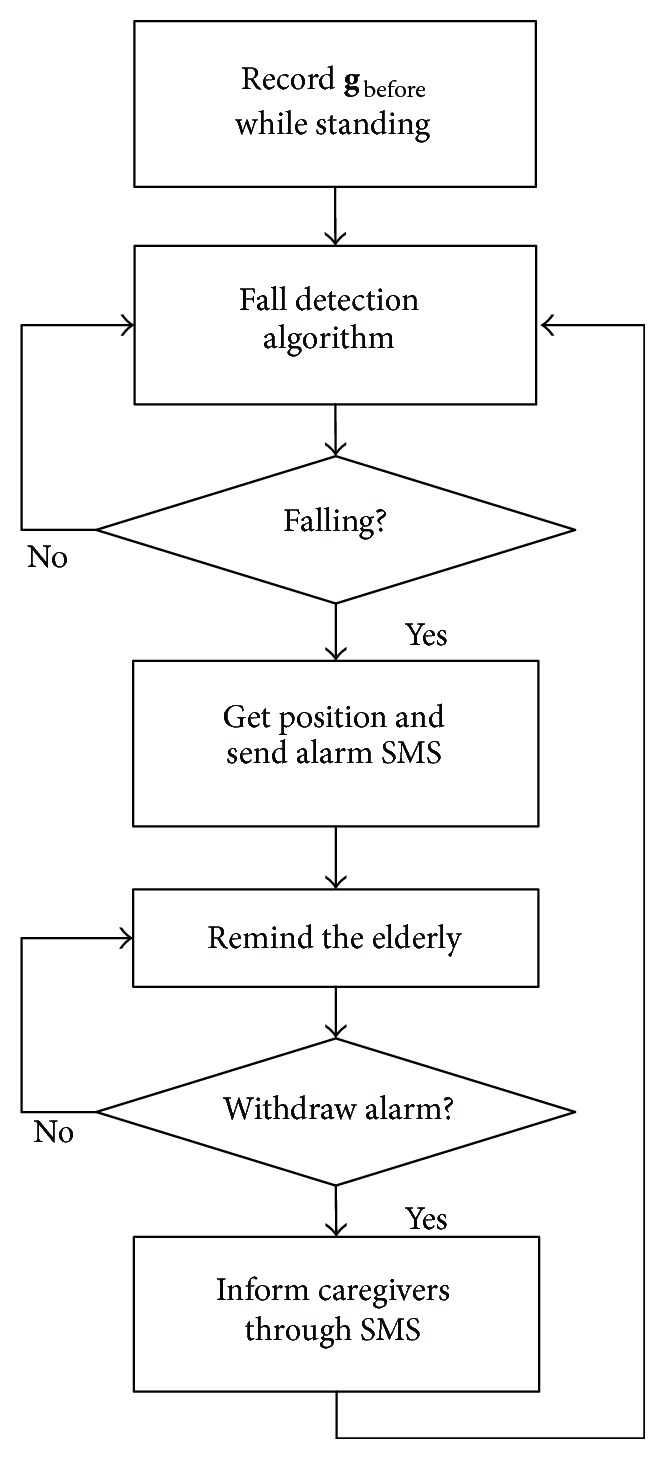
System software flow diagram.

Flow diagram of the wearable device software is shown in Figure 8.

After initialization of the system, **g**_before_ would be extracted from acceleration measurements by using a low pass filter when the elderly is standing. After that, the fall detection algorithm will be applied. If a fall has been detected, the wearable device will locate the user and send alarm short message to caregivers immediately. Then the device will remind the elderly through vibration. If the user withdraws the alarm by pressing a button manually, the device will get back to fall detection state and a short message will be sent to inform the caregivers.

#### 4.2.2. Caregiver's Handset

There is a URL (universal resource locator) which links to a web map in the alarm short message. The fall location information will be highlighted on a web map when the caregiver opens this link.

## 5. System Test

System test of the fall detection system has been conducted based on the system design described above.

### 5.1. Wearable Device and Fall Detection Algorithm

The sampling rate of accelerometer is set at 100 Hz and the measurement range is ±16 g with a maximum precision of 4 mg. MCU will read raw measurements from sensor's inner FIFO and apply the detection algorithm.

The test objects are three different volunteers at the ages of 23, 42, and 60, respectively. Based on analysis of these volunteers' experiment data, *a*_threshold_ and *t*_threshold_ are set as 2 g and 2 s, respectively. In order to get **g** when standing and lying after the fall, sum acceleration **a** which has magnitude between (1 − 0.3) g and (1 + 0.3) g will be considered as **g**_before_ and **g**_after_. Considering that the tilt of the ground or the lying posture of the elderly may affect the rotation angle, rotation angle *θ* between (90 − 30)° and (90 + 30)° will infer that the elderly has fallen.

System test contains five kinds of activities of daily living (i.e., walking, jumping, squatting, sitting, and resting) and four kinds of fallings (i.e., forward, backward, leftward, and rightward).

Each kind of motion has been repeated 20 times on each volunteer, and the detection results of the proposed algorithm and an acceleration threshold based algorithm [13] are listed in Table 1.

The sensitivity and specificity [21, 22] of the proposed system can be got from the test data in Table 1. Sensitivity of the proposed algorithm is 97.1%, and the specificity is 98.3%. Test results of acceleration threshold based algorithm show lower sensitivity and specificity at 91.6% and 88.7%, respectively.

**Figure 9:**
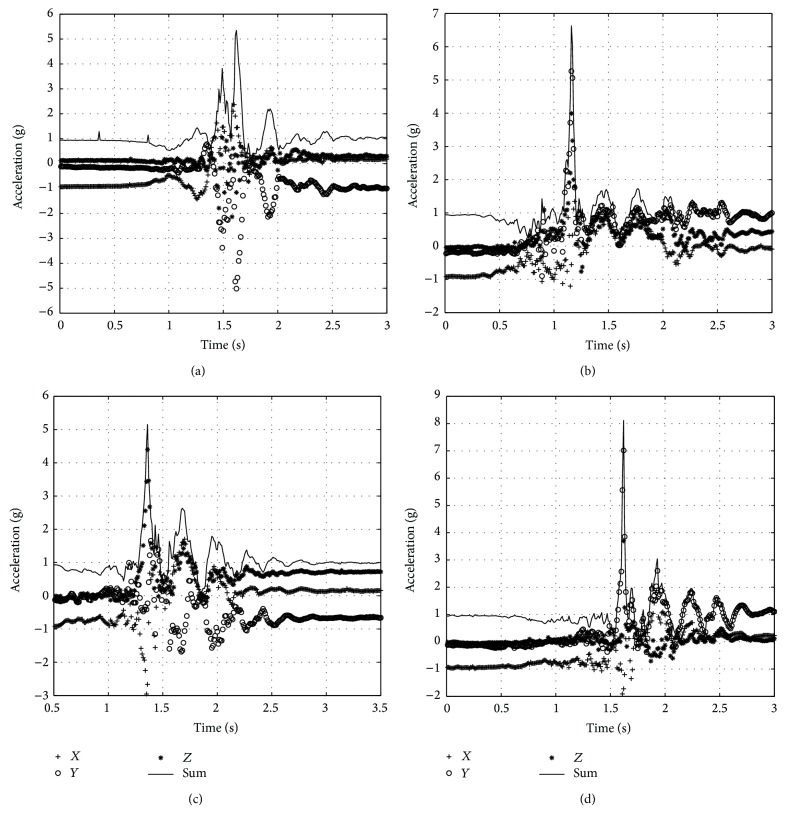
Acceleration waveforms during fall. (a) Forward fall. (b) Backward fall. (c) Leftward fall. (d) Rightward fall.

Figure 9 shows the accelerations in different kinds of fallings which all trigger the fall alarm correctly and the corresponding rotation angles are 91.9°, −100.5°, 104.7°, and −114.1°.

**Figure 10:**
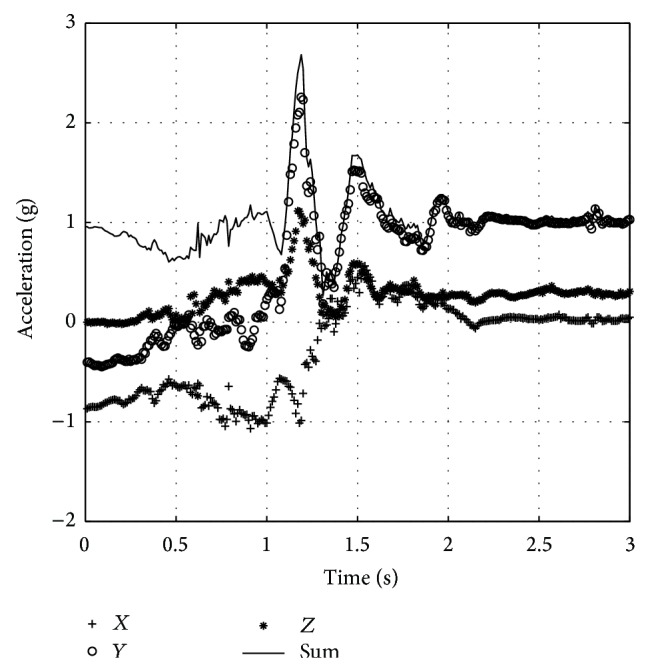
Resting.

Figure 10 shows acceleration during activity of lying down and resting which has rotation angle of −117.0° and similar waveform to falling [23], but the peak acceleration is much lower. Fall alarm has not been triggered yet at this situation.

**Figure 11:**
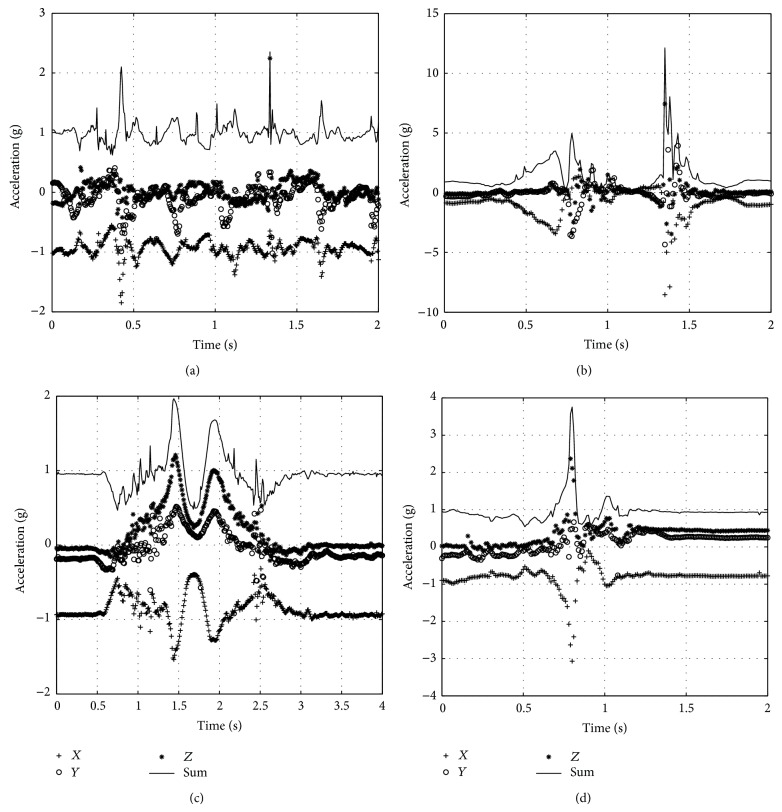
Acceleration waveform during ADL. (a) Walking. (b) Jumping. (c) Squatting. (d) Sitting.

Figure 11 shows acceleration during other ADL tests. They all do not have rotation about 90°; even some of the peak values are quite high, so the fall alarm has not been triggered.

### 5.2. Location Based Service

**Figure 12:**
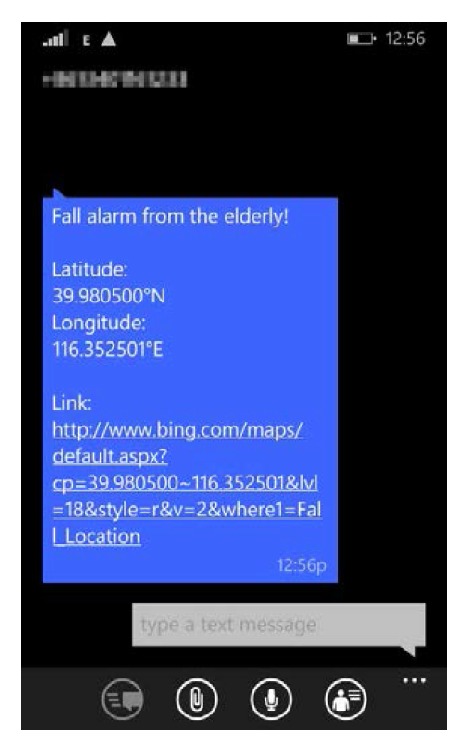
Fall alarm SMS which contains fall location URL.

**Figure 13:**
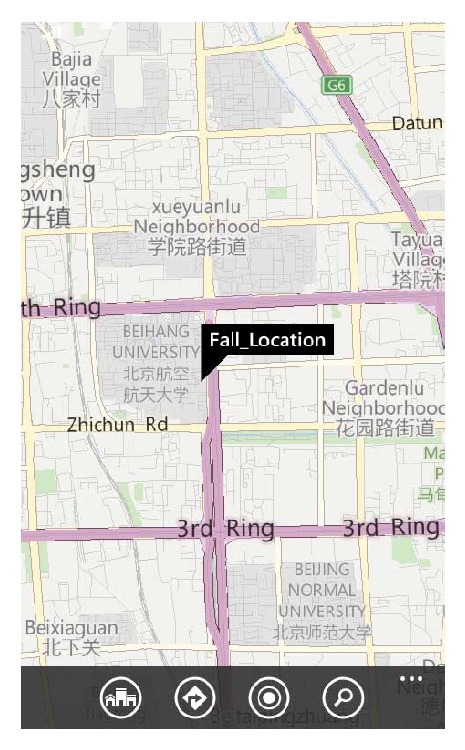
Fall location displayed on a Web map.

Alarm receiving function on caregiver's handset has also been tested. An alarm SMS (short message service) containing a map URL has been received by the handset as shown in Figure 12 when a fall has been detected. Clicking the URL will open a map in Web browser on which the fall location will be displayed accurately as shown in Figure 13.

## 6. Conclusion

This paper developed a fall detection system based on a single triaxial accelerometer based wearable device. There is no special requirement of the device's mounting orientation because the algorithm does not claim the axes of accelerometer to be fixed strictly. The system has low power consumed hardware design and highly efficient algorithm which could extend the service time of the wearable device. Both the hardware and software designs are suitable for wearable and outdoor application.

As normal activity of resting also has similar rotation as falling, it may trigger fall alarm when the body hits ground heavily. So the choice of *a*_threshold_ is quite important to distinguish falling from heavily lying activity. Sufficient sample number collected from subjects with different age and gender will improve the reliability and robustness of the threshold. Beside these, technologies such as SVM and neural network are considerable to seek out a proper classification method based on the features used in this system.

## Figures and Tables

**Table 1:** Test results for four kinds of falling and five kinds of ADL.

Motion type	Alarm times/test times	
	Proposed design	Acceleration threshold based design
Forward fall	60/60	59/60
Backward fall	56/60	54/60
Leftward fall	58/60	52/60
Rightward fall	59/60	55/60
Walking	0/60	4/60
Jumping	0/60	11/60
Squatting	0/60	0/60
Sitting	0/60	9/60
Resting	5/60	10/60
